# Injectable, Biodegradable Hydrogels for Tissue Engineering Applications

**DOI:** 10.3390/ma3031746

**Published:** 2010-03-10

**Authors:** Huaping Tan, Kacey G. Marra

**Affiliations:** 1 Division of Plastic Surgery, Department of Surgery, University of Pittsburgh, Pittsburgh, PA, USA; E-Mail: tanh@upmc.edu (H.T.); 2 Department of Bioengineering, University of Pittsburgh, Pittsburgh, PA, USA; 3 McGowan Institute for Regenerative Medicine, University of Pittsburgh, Pittsburgh, PA, USA

**Keywords:** injectable hydrogels, biodegradation, biomaterials, tissue engineering, regeneration medicine

## Abstract

Hydrogels have many different applications in the field of regenerative medicine. Biodegradable, injectable hydrogels could be utilized as delivery systems, cell carriers, and scaffolds for tissue engineering. Injectable hydrogels are an appealing scaffold because they are structurally similar to the extracellular matrix of many tissues, can often be processed under relatively mild conditions, and may be delivered in a minimally invasive manner. This review will discuss recent advances in the field of injectable hydrogels, including both synthetic and native polymeric materials, which can be potentially used in cartilage and soft tissue engineering applications.

## 1. Introduction

**Figure 1:**
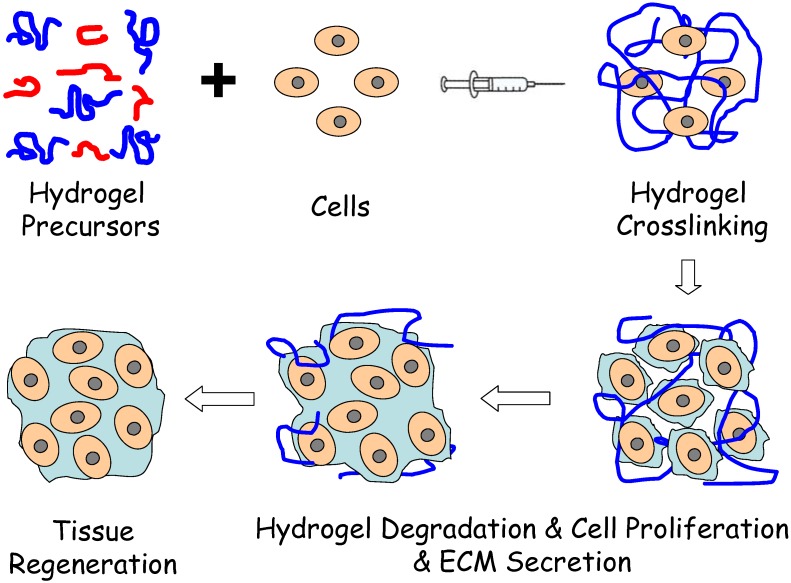
Schematic illustration of injectable hydrogel for tissue regeneration approaches. Cells are isolated from a small biopsy, expanded *in vitro*, and encapsulated in hydrogel precursors, which are subsequently transplanted into the patient by injection using a needle. Hydrogel provide the initial structural support and retain cells in the defective area for cell growth, metabolism and new ECM synthesis. The hydrogel is readily degradable when the cells secrete ECM. This strategy enables the clinician to transplant the cell, growth factor and hydrogel combination in a minimally invasive manner.

Injectable hydrogels are promising substrates for tissue engineering applications due to high tissue-like water content, ability to homogeneously encapsulate cells, efficient mass transfer, easily manipulated physical properties and minimally invasive delivery [1,2,3]. The hydrogel precursor loaded with growth factors and/or targeted cells can be injected into the wound site and experiences a solution-to-gelation transition (sol–gel) *in situ* due to physical or chemical stimuli [4,5,6]. The injectable nature of the hydrogels provides the attractive feature of facile and homogenous cell distribution within any defect size or shape prior to gelation. Highly hydrated hydrogels can better mimic the chemical and physical environments of ECM and therefore are ideally cellular microenvironment for cell proliferation and differentiation. Most importantly, injectable hydrogels have a similar microstructure to the extracellular matrix (ECM) and allow good physical integration into the defect, potentially avoiding an open surgery procedure and facilitating the use of minimally invasive approaches for material and cell delivery [7,8]. The encapsulated cells grow within the hydrogel and secrete new ECM to restore the damaged tissue [9]. Figure 1 depicts this process.

Over the past decade, a variety of naturally- and synthetically-derived materials have been utilized to form injectable hydrogels for tissue engineering applications [1,2,5]. Natural polymers have been widely used as hydrogels for tissue engineering approaches due to excellent biocompatibility. A wide range of synthetic hydrogels may potentially have suitable physical and chemical properties for tissue engineering applications. However, synthetic polymers may lack informational structure for positive cell biological response. As a consequence, modification of synthetically derived hydrogels is usually required. The reported injectable and biodegradable hydrogels systems derived from natural and synthetic materials are listed in the Table 1.

Many methods have been employed for preparation of injectable *in situ* forming hydrogels, including thermal gelation, ionic interaction, physical self-assembly, photopolymerization and chemical crosslinking with agents such as glutaraldehyde, genipin, adipic dihydrazide and bis(sulfosuccinimidyl) suberate [5,6,7]. Injectable hydrogels with potential applications in tissue engineering can be classified into physical and chemical gels, according to their gelation mechanism [10]. The hydrogel network crosslinked by physical association between polymeric chains or nanoparticles is the so-called physical gel, while the formation of a chemical gel takes place *via* covalent bonds between polymeric chains. Conventional hydrogel synthesis methods are limited by operational complexity, involvement of cytotoxic reagents, instability of the functional groups, possible side reactions and low coupling efficiency. There is a continuing need to exploit simple, specific, and highly efficient conjugation methods which are applicable to a broad class of biodegradable hydrogels with full preservation of bioactive function for tissue engineering.

**Table 1:** Injectable and biodegradable hydrogels for tissue engineering.

Hydrogels	Polymers	Gelation Mechanism
Natural hydrogels	Collagen/Gelatin	Thermal/Chemical crosslinking
	Chitosan	Thermal/Chemical/Schiff-base reaction/Free radical crosslinking
	Hyaluronic acid	Thermal/Chemical/Schiff-base reaction/Michael-type addition/Free radical crosslinking
	Chondroitin sulfate	Free radical crosslinking
	Alginate	Ionic/Free radical crosslinking
	Agar/Agarose	Thermal crosslinking
	Fibrin	Thermal crosslinking
Synthetic hydrogels	PEG/PEO	Michael-type addition/Chemical/Free radical crosslinking
	PVA	Chemical/Free radical crosslinking
	PPF/OPF	Free radical crosslinking
	PNIPAAm	Thermal crosslinking
	PEO-PPO-PEO PLGA-PEG-PLGA PEG-PLLA-PEG	Thermal crosslinking
	Poly(aldehyde guluronate)	Chemical crosslinking
	Polyanhydrides	Free radical crosslinking

To develop a suitable hydrogel as a cell carrier, the degradation rate and mechanical properties of the hydrogel must complement the tissue growth and natural ECM. In general, these properties can be fine-tuned through variations in the chemical structure and crosslinking density in hydrogels. For a given hydrogel system, activities of seeded cells can be regulated by attaching specific bioactive moieties to the polymer matrix backbone. Comprised of various ECM-like macromolecules and proteins, hydrogels control the tissue structure, regulating the function of the cells. In this review, we will discuss the structure design of injectable and biodegradable hydrogel systems to be potentially used in cartilage and soft tissue (e.g., adipose) engineering applications.

## 2. Biodegradable Materials for Injectable Hydrogels

Natural biomaterials exhibit excellent bioactivity due to their components of ECM, thus containing cell-specific domains such as RGD (Arg-Gly-Asp) sequence. However, hydrogels derived from natural polymers often undergo rapid degradation upon contact with body fluids or medium. Therefore, limitations of natural hydrogels have motivated approaches to modify these polymers as well as to utilize various synthetic polymers. An appealing and effective strategy is to incorporate bioactive species such as cells, growth factors, peptides and proteins into the material, resulting in biomimetic hydrogel scaffolds with bioactive functions for optimal cell response.

### 2.1. Natural Materials

Naturally-derived hydrogel forming polymers have frequently been used in tissue engineering applications because they are either components of or have macromolecular properties similar to the natural ECMs [11,12]. Representative naturally derived polymers include collagen, gelatin, chitosan, hyaluronic acid, chondroitin sulfate, agarose, alginate, and fibrin.

Collagen is an attractive material for biomedical applications as it is the main component of natural ECM and the most abundant protein in mammalian tissues [13,14]. The basic structure of collagen is composed of three polypeptide chains, which wrap around one another to form a three-stranded rope structure [5,14,15]. Collagen is naturally degraded by metalloproteases, specifically collagenase, and serine proteases [16,17], allowing for degradation to be locally controlled by cells present in the engineered tissue. Gelatin is a partial derivative of collagen, formed by breaking the natural triple-helix structure of collagen into single-strand molecules by hydrolysis [18]. Gelatin is less immunogenic compared to its precursor and presumably retains informational signals such as the RGD sequence, thus promoting cell adhesion, migration, differentiation and proliferation [19]. Collagen and gelatin hydrogels can be formed and their mechanical properties enhanced by introducing various chemical crosslinkers (*i.e.*, glutaraldehyde, genipin and carbodiimide).

Chitosan is a linear polysaccharide, which is a partially deacetylated derivative of chitin. This polycationic polysaccharide contains glucosamine and *N*-acetylglucosamine molecules, thus structurally similar to naturally occurring glycosaminoglycans (GAGs) [20,21,22]. Chitosan is considered a biodegradable polysaccharide, which can be metabolized by human enzymes such as lysozyme [23,24]. Chitosan has been investigated for a variety of tissue engineering applications in recent years due to its biocompatibility, biodegradability, low immunogenicity and cationic nature [22,23,24,25]. However, unmodified chitosan can only be dissolved in acidic solutions due to its strong intermolecular hydrogen bonds, which limits its applications as an *injectable* hydrogel. Water-soluble chitosan derivatives support cell growth, and composites of chitosan and GAG or other bioactive proteins are able to create suitable biomimetic microenvironments for cell implantation [21,22,23]. Chitosan derivatives have been gelled *via* glutaraldehyde crosslinking, UV irradiation, and thermal variations. Recently, chitosan molecules were also grafted with poly(*N*-isopropylacrylamide) (PNIPAAm) and *N*-isobutyryl groups to obtain a thermosensitive chitosan hydrogel, or vanillin or hydroxybenzaldehydes to obtain an ultraviolet (UV) crosslinkable chitosan hydrogel with improved biocompatibility [21,22,23,24,25,26].

Hyaluronic acid (HA) is a naturally occurring non-sulfated glycosaminoglycan that is widely distributed throughout the ECM of all connective tissues in human and other animals [27,28]. Hyaluronic acid plays an essential role in many biological processes such as tissue hydration, nutrient diffusion, proteoglycan organization, and cell differentiation. HA is especially prevalent during wound healing and in the synovial fluid of joints. Hyaluronic acid is a GAG consisting of multiple repeating disaccharide units of *N*-acetyl-*D*-glucosamine and *D*-glucuronic acid. Hyaluronic acid is naturally degraded by hyaluronidase [29], which is ubiquitous in cells and in serum. Due to its good biocompatibility, biodegradability, as well as excellent gel-forming properties, HA and its derivatives have been widely explored as hydrogels for tissue engineering [30,31]. Hyaluronic acid hydrogels can be formed by covalent crosslinking with hydrazide derivatives, esterification, and annealing [30,31,32,33,34,35]. Additionally, hyaluronic acid has been combined with both collagen and alginate to form composite hydrogels [33,34,35].

Alginate is a hydrophilic and linear polysaccharide composed of (1–4)-linked *β*-D-mannuronic acid (M) and *α*-L-guluronic acid (G) monomers, which are derived primarily from brown seaweed and bacteria [1,2,36,37,38]. Simple gelation can be formed when divalent cations such as Ca^2+^, Mg^2+^, Ba^2+^, or Sr^2+^ cooperatively interact with blocks of G monomers to form ionic bridges [5,39,40,41]. Alginate has been used in a variety of medical applications including cell encapsulation, tissue engineering and drug delivery, because it gels under gentle conditions, has low toxicity, and is readily available [42,43,44,45,46]. Despite its advantageous features, alginate may not be an ideal candidate for tissue engineering because it does not specifically degrade. Ionically crosslinked alginate hydrogel degrades *via* an ion exchange process involving loss of divalent ions into the surrounding medium, and undergoes an uncontrolled dissolution. Alginate has been covalently coupled with lectin and RGD to enhance cell ligand-specific binding properties due to lack of cellular interaction in its molecular structure for tissue engineering applications [1,5,44,45].

### 2.2. Synthetic Materials

Synthetic polymers are appealing for hydrogels because their chemical and physical properties are typically more controllable and reproducible than those of natural polymers. Synthetic polymers can be reproducibly produced with specific block structures, molecular weights, and degradable linkages. Compared to natural hydrogels, synthetic hydrogels offer improved control of the matrix architecture and chemical composition, but tend to have lower biological activity. One approach to creating an ideal hydrogel for tissue engineering applications is to incorporate bioactive elements into synthetic hydrogels for increased cellular bioactivity. Synthetically-derived materials include poly(ethylene glycol) (PEG), poly(vinyl alcohol) (PVA), poly(propylene fumarate) (PPF), PNIPAAm, Pluronic F-127 and polypeptides, which are among the most widely used synthetic polymers for injectable hydrogels.

PEG is currently FDA-approved for several medical applications. Although many variations of synthetic biocompatible, biodegradable polymers can form hydrogels *via* chemical crosslinking, PEG remains one of the most widely investigated systems [47,48,49,50]. Biodegradable PEG hydrogels can be obtained *via* copolymerization with degradable polymers such as poly(lactic acid), poly(glycolic acid) and poly(propylene fumarate) [51,52,53]. Furthermore, many naturally occurring biopolymers, such as hyaluronic acid, fibrinogen, chitosan, and heparin, are also generally examined in combination with biodegradable PEG hydrogels [54,55,56,57,58]. PEG hydrogels have been used as cell scaffolds, adhesive medical applications, and delivery vehicles with promising results [59,60,61,62]. Particularly, the ability to control the crosslinking density provides the flexibility and tailorability to PEG-based hydrogels for cell encapsulation and tissue growth. PEG and the chemically similar poly(ethylene oxide) (PEO) are hydrophilic polymers that can be photocrosslinked by modifying each end of the polymer with either acrylates or methacrylates.

PVA and PPF are also synthetic hydrophilic polymers that have been widely explored for injectable hydrogel using in tissue engineering applications. PVA can be modified into multifunctional macromers through the plethora of pendant hydroxy groups, which can be derivatized with a variety of substituents [5]. It can be physically and chemically crosslinked to form hydrogels as well as blended with other water-soluble polymers [2,63,64]. PVA hydrogels can be formed by physically crosslinking through repeated freezing/thawing methods, or chemically crosslinked with glutaraldehyde or epichlorohydrin [1,5]. PPF is a linear polyester, which undergoes degradation by hydrolysis of the ester linkage. PPF can form hydrogels when synthesized as a block copolymer with hydrophilic PEG and crosslinked *via* UV exposure and chemically [1,2].

## 3. Injectable Hydrogel Systems

Many methods have been employed for preparation of injectable hydrogels. Among these gelation methods, thermal crosslinking is relatively easy without limitation of the injection depth, as is a concern with photopolymerization. In many of the natural hydrogels, e.g., collagen and fibrin glue, the physical and ionic crosslinking mechanisms are difficult to control, which limit the final network structure and properties. In contrast, covalently crosslinked hydrogels offer many advantages such as controllable crosslinking density and structure properties. Particularly, the ability to control the crosslinking density provides the flexibility to design a wide range of polymeric networks for cell encapsulation and tissue growth.

### 3.1. Physical Crosslinking of Hydrogels

Biodegradable hydrogels capable of phase transition in response to external stimuli such as temperature represent another method of preparing injectable hydrogels for biomedical applications. Thermoresponsive phase transition has been utilized for potential tissue regeneration because gelation can be realized simply as the temperature increases above the lower critical solution temperature (LCST), which is designed to be below body temperature.

Recently, a pH-neutral chitosan solution was developed by the addition of a polyol counter-ionic dibase salt such as *β*-glycerol phosphate disodium. Chenite *et al*. [65] demonstrated that chitosan solution neutralized with *β*-glycerol phosphate disodium form *in situ* gelling systems, which remain liquid for long periods at room temperatures but are transformed into a macroporous gel as temperature raised to 37 °C. Solubility of chitosan in aqueous solutions is attained *via* protonation of its amine groups in acidic environments. Neutralization of chitosan aqueous solutions to a pH exceeding 6.2 systematically leads to the formation of a hydrated gel-like precipitate. The combination of chitosan and *β*-glycerol phosphate disodium benefit from several synergistic forces favorable to gel formation including hydrogen bonding, electrostatic interactions and hydrophobic interactions [65]. The uniqueness also resides in overcoming this pH barrier for chitosan solutions, which has long been a major limitation for many applications. This quality allows the material to be injected and to form a scaffold [66,67] *in situ* with minimal surgical destruction. Injection and cultivation of this hydrogel loaded with chondrocytes in a mouse model formed a proteoglycan-rich matrix *in vivo*. These systems gelled at body temperature, retained their physical properties over a long period depending on the storage conditions, and sustained the release of macromolecules over few hours to a few days [67,68].

**Figure 2:**
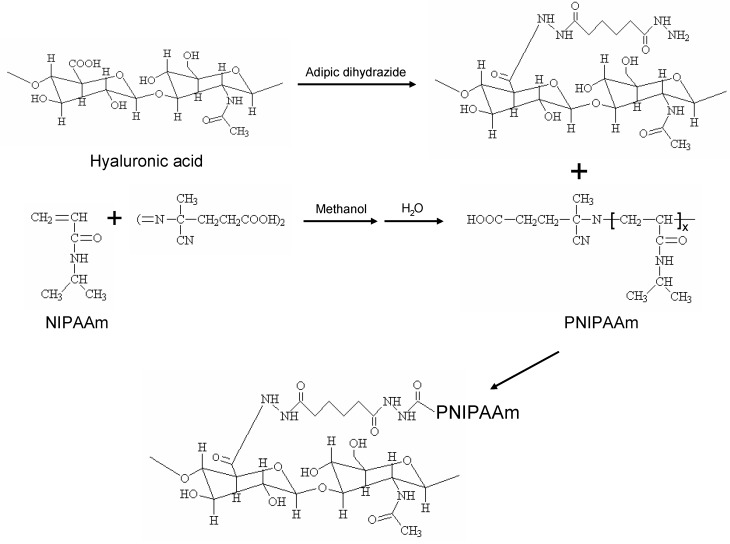
Synthetic route of PNIPAAm-grafted aminated hyaluronic acid (AHA-g-PNIPAAm).

PNIPAAm is an example of thermosensitive polymers that undergo a coil-to-globule phase transition ~32 °C [69,70,71,72,73,74,75]. The main mechanism of the aqueous phase separation of PNIPAAm is the thermally induced release of water molecules bound to the polymer isopropyl side groups, which results in increasing intra- and inter-molecular hydrophobic interactions between the isopropyl groups above its LCST [76,77,78]. The thermosensitivity of hydrogels can be achieved by incorporating PNIPAAm into the backbone of biodegradable polymers. Over the past decade, various thermosensitive and injectable polymers including PEG, chitosan, gelatin, hyaluronic acid, and PNIPAAm copolymers have been developed and employed in a variety of settings [71,72,73,74,75,76,77,78,79,80,81,82,83,84]. An effective method to combine the PNIPAAm with biodegradable polymers is copolymerization by free radical polymerization using 2,2-azobisisobutyronitrile (AIBN), 4,4’-azobis(4-cyanovaleric acid) (ACA), benzyl peroxide (BPO) and ammonium persulfate (APS) as initiators [76,77,78,79]. The procedure involves the synthesis of a carboxyl- or amino-terminated NIPAAm copolymer, which is then coupled onto biomacromolecular or peptide sequences. For example, hyaluronic acid was aminated and partially grafted with PNIPAAm-COOH (Figure 2), in which the desired degree of grafting are obtained under mild reaction conditions. The potential application of the thermoresponsive hyaluronic acid as a functional injectable scaffold in soft tissue engineering was studied by encapsulation behavior of human adipose-derived stem cells (ASCs). A preliminary *in vitro* and *in vivo* study indicates that the thermosensitive hyaluronic acid copolymer hydrogel with 53% PNIPAAm may have potential uses in adipose regeneration and other soft tissue engineering applications.

Triblock copolymers have been widely studied by many researchers in injectable cell delivery systems as an inverse thermogelling polymer by micelle formation. The block copolymers such as PEO-PPO-PEO (Pluronic), PLGA-PEG-PLGA, PEG-PLLA-PEG, PCL-PEG-PCL, PCLA-PEG-PCLA and PEG-PCL-PEG are typical thermosensitive biodegradable polymers exhibiting sol-gel transitions in water with increase of temperature [85,86,87,88]. The amphiphilic block polymer chains assemble first into micelles and bridged micelles at low temperatures, and then the ordered packing of bridged micelles is triggered at a higher temperature and a macroscopic gel forms [85,86]. These thermogelling copolymer hydrogels have been successfully applied in cell therapy, tissue regeneration, and wound healing due to their biocompatibility and long persistence in the gel form *in vivo*.

In addition to the traditional hydrogels, with smart response to pH, temperature, ions, and stresses, self-assembled hydrogels containing peptide domains present a novel advance in terms of their structural similarity to natural ECM, and their bioactivity to cartilage and soft tissue regeneration have been reported [89,90]. Some hydrogels can be crosslinked by interaction between or among self-assembled *α*-helix structures, and others may self-assemble to form nanofibrous structures such as DNA sequences and molecules [91].

### 3.2. Chemical Crosslinking of Hydrogels

#### 3.2.1. Free radical polymerization

Photopolymerization is a process that enables *in situ* formation of crosslinked networks at physiological pH and temperature [92,93]. The mild gelation conditions allow for cells to be encapsulated within photocrosslinked hydrogels and remain viable. The unique advantage of chain polymerization is the ease with which a variety of chemistries can be incorporated into the hydrogel by simply mixing derivatized macromers of choice and subsequently copolymerizing [93,94,95]. Furthermore, photopolymerized hydrogel systems can provide better temporal and spatial control over the gelation process, are injectable in nature, and can polymerize *in situ* to fill defects of any shape.

Many researchers are interested in exploiting the photoinitiated polymerization of PEG-based macromolecular monomers to create hydrogels as cell delivery vehicles for tissue regeneration [96,97,98,99]. An extensively studied PEG hydrogel system is to employ ultraviolet irradiation to generate radicals from appropriate photoinitiators, which will further react with the active end group on modified PEG to form a covalent crosslinked bond. Poly(ethylene-glycol)-diacrylate (PEGDA) and poly(ethylene glycol)-dimethacrylate (PEGDMA) are modified PEGs with unsaturated C=C double bond groups, rendering them as photopolymerizable hydrogel candidates for UV exposure. Previous studies have demonstrated that PEGDA and PEGDMA can be used to photoencapsulate chondrocytes and bone marrow stromal cells (MSCs) [98,99,100,101]. Cell suspensions in PEGDA and PEGDMA solution can be injected into the body and be polymerized with UV exposure to form a crosslinked PEG gel that functions as a tissue scaffold. Furthermore, copolymerization of PEG with other synthetic macromers such as PVA enables additional control of functionality and properties that are especially important from a tissue engineering perspective. For cell delivery purposes, PEG is composed of a biochemically inert polymer that lacks the ability to adhere to cells. Hybrid artificial scaffolds that combine the physical characteristics of the PEG gels and bioactive features of natural collagen in the hopes of creating a scaffold, which is photopolymerizable and at the same time provides an ideal microenvironment for encapsulated cells. In addition to PEG, alginate, chitosan, hyaluronic acid and chondroitin sulfate were also methacrylated and hydrogels were prepared by photopolymerization and other free radical polymerizations [98,99,100,101,102,103,104].

Photo-crosslinkable poly(propylene fumarate) (PPF) and oligo(poly(ethylene glycol) fumarate) (OPF) hydrogels have been extensively developed for use in tissue engineering applications [105,106,107,108,109,110,111]. The mechanical properties and degradation rates of the PPF and OPF hydrogels are controlled in macromer formation as both are biodegradable. PPF and OPF macromers are composed of biocompatible blocks such as PEG and fumaric acid, which can be crosslinked through the unsaturated C=C double bond in the fumarate group and hydrolytically degraded through its ester bonds. In addition, at neutral pH and body temperature, the three-dimensionally crosslinked PPF and OPF hydrogels have been formed by polymerization of the C=C bonds under the initiation of a redox system, ammonium persulfate (APS)/*N,N,N’,N’*-tetramethylethylenediamine (TMEDA). Analyzed by *in vitro* cytotoxicity assay and *in vivo* implantation, the PPF and OPF hydrogels have shown minimal or negligible cytotoxicity and is histocompatible. Therefore, these materials can provide appropriate properties for an ideal injectable cell carrier.

#### 3.2.2. Michael-type addition reaction hydrogels

Michael-type conjugate addition reaction can be used for the gelation of injectable hydrogels. For example, the thiol and vinylsulfone on peptides or polymeric macromolecules such as hyaluronic acid can conjugate and form synthetic extracellular matrices as hydrogels for tissue engineering [112,113]. Vinylsulfones with low molecular weights are known to demonstrate toxicity because they can easily enter into the cell’s cytoplasm, resulting in toxic reactions with glutathione and DNA [114]. An effective method to prevent the toxicity of small vinylsulfone molecule is coupling to the backbone of watersoluble biomacromolecules. For example, *in situ* crosslinkable hydrogels based on thiol-modified hyaluronic acid satisfy many of the design criteria for *in vitro* and *in vivo* tissue engineering [114,115,116,117]. It was shown that cells can survive polymerization of hydrogel and maintain their viability in hyaluronic acid hydrogels containing covalently linked gelatin or RGD peptides. In addition, Michael-type conjugate addition reaction has been applied to the methacrylate HA-based hydrogels, but a longer gelation time is required for hydrogel formation due to its relatively lower reactivity of the methacryl group. To further accelerate the gelation of the hydrogel for cell delivery procedure, an alternative system based upon the conjugate addition crosslinking between thiol-modified HA and PEGDA was developed, which satisfies most key requirements for injectable *in vivo* tissue engineering applications [115,116].

**Figure 3:**
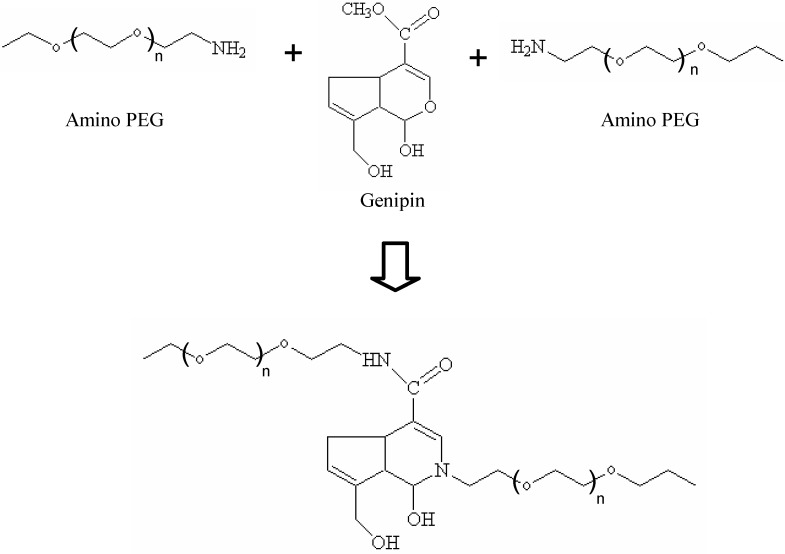
The reaction scheme of amino PEG-genipin hydrogel.

#### 3.2.3. Genipin crosslinked hydrogels

Genipin is a natural product extracted from the gardenia fruit, which overcomes the toxicity inherent in most commonly used synthetic cross-linkers [118,119]. Recent studies identified that genipin can be utilized to crosslink functional amine groups present in natural tissues and biomaterials with very minimal cytotoxic effects, compared to studies performed with glutaraldehyde, a commonly used crosslinker [119,120,121,122,123], resulting in materials with a deep blue color. The utilization of genipin (0.5~3.5 wt %) to crosslink natural biocompatible polymers, such as chitosan and gelatin, to form biodegradable hydrogels has the potential to produce novel scaffolds for various tissue engineering applications. Our laboratory has recently examined the synthesis of novel amino-terminated multi-arm PEG based hydrogels utilizing genipin as a crosslinking agent [124,125,126,127]. We examined 2 PEG structures: 4-arm PEG, a molecule with 4 PEG chains attached at a central point, and 8-arm PEG, a molecule with 8 PEG chains attached to a central molecule (Figure 3). The gelation time, swelling, water uptake and weight loss were dependent on the structure of the PEG hydrogel. Due to the molecular architecture, the 8-arm PEG hydrogel showed a much slower gelation reaction, more compact structure and lower water uptake than those of the 4-arm PEG hydrogel, as well as a better stability *in vitro* with a 35.2 mM genipin. Furthermore, human adipose derived stem cell study results indicated that both the 4-arm and 8-arm PEG hydrogels are able to support cell adhesion. This study represents the potential opportunity to use genipin cross-linked, multi-arm PEG-genipin hydrogels, especially the 4-arm PEG-genipin, as an injectable scaffold in a variety of tissue engineering applications.

#### 3.2.4. Schiff-base crosslinked hydrogels

**Figure 4:**
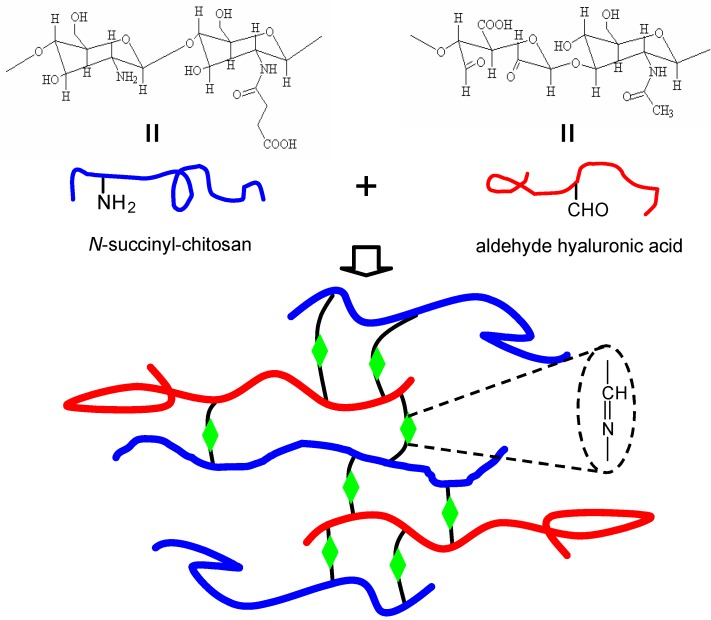
The scheme of *N*-succinyl-chitosan and aldehyde hyaluronic acid composite hydrogel *via* Schiff’s base cross-linking reaction.

More recently, we have developed a new injectable, *in situ* forming biocompatible and biodegradable hydrogel as cell carriers for tissue engineering applications [128]. The polysaccharide hydrogel derived from water-soluble chitosan and oxidized hyaluronic acid gel upon mixing, without employing any extraneous chemical crosslinking agents. The gelation is attributed to the Schiff-base reaction between amino groups of *N*-Succinyl-chitosan and aldehyde groups of oxidized hyaluronic acid (Figure 4). *N*-Succinyl-chitosan, a water soluble chitosan derivative, was synthesized *via* introduction of succinyl groups at the *N-*position of the glucosamine units of chitosan. Hyaluronic acid can be oxidized, and the carbon-carbon bonds of the cis-diol groups in molecular chain are cleaved and generate reactive aldehyde functions, which can develop chemical crosslinking action with amino functions *via* Schiff-base linkage. This polysaccharide hydrogel creates a biomimetic microenvironment with improved biocompatibility and biodegradation for tissue regeneration. Several other polysaccharides such as dextran, gum arabic and chondroitin sulfate can be partially oxidized and employed for Schiff-base linkage [129,130,131,132,133,134].

## 4. Applications of Injectable Hydrogels

### 4.1. Clinical Applications

The need for injectable, biodegradable hydrogels in biomedical applications is immense. One example is the utility of hydrogels in cartilage regeneration. The physical properties of the hydrogel can be designed to easily match those of articular cartilage in addition to matching mechanical properties of the scaffold with the native tissue. Further applications for hydrogels include soft tissue regeneration after tumor removal or trauma. A number of researchers have studied the combination of injectable hydrogels and biodegradable microspheres for controlled drug delivery in tissue engineering, including our own laboratory [135,136,137,138,139]. The following sections describe the pre-clinical and clinical studies of hydrogels for these applications.

### 4.2. Cartilage Repair

The need for tissue-engineered cartilage is immense and of great clinical significance. Traumatic and degenerative lesions of articular cartilage are leading causes of disability [140]. It is estimated that over 40 million Americans currently suffer from osteoarthritis [141]. Tissue engineering methods, including the use of injectable hydrogels, to improve cartilage repair and regeneration will therefore have high clinical impact. The advantage of injectable therapies for cartilage repair is that the implant is not only maintained within the defect, but also allows immediate weight-bearing due to the stiffness and strength that is achieved almost instantly. Additionally, a general advantage of injectable therapies is the utilization of minimally invasive surgery as compared to open surgery. As such, there have been numerous studies involving the use of injectable hydrogels for cartilage repair.

### 4.3. Soft Tissue Regeneration

Soft tissue reconstruction is a significant challenge in reconstructive surgery. There are several reasons for a lack of soft tissue, e.g., adipose tissue, such as congenital (e.g., in Parry-Romberg syndrome [142] or Poland syndrome [143], both of which can result in lipoatrophy), traumatic, or oncologic surgery. Due to a lack of better alternatives, transplantation of autologous adipose tissue has been used for soft tissue reconstruction for the past century. However, the clinical outcome of adipose tissue transplantation is unpredictable as there is variable graft resorption due to a lack of vascularization [144]. A desirable strategy to repair soft tissue is to induce adipogenesis *in situ*. One method to accomplish this is to utilize cells that can differentiate to form adipose tissue, and seed those cells into a scaffold, resulting in adipose tissue formation. Another strategy is to utilize injectable systems. As such, many injectable hydrogels based on both synthetic and natural biomaterials have been examined. For example, Hemmerich *et al*. reported the reconstruction of small defects using injectable hyaluronic acid-based gel which were mixed with undifferentiated adipose-derived stem cells (ASCs). Adequate adipose tissue formation was observed using ASCs and hyaluronic acid as the scaffold [145]. Hyaluronic acid, therefore, is applicable for generating adipose tissue in gels, displaying adipogenic as well as angiogenic properties [146].

Other injectable scaffold matrices include biodegradable, polymeric microspheres. For example, Yuksel *et al*. reported the release of insulin-like growth factor-1 (IGF-1) as well as insulin from PLGA microspheres enhanced *de novo* adipose tissue formation [147]. Their study demonstrated the potential of long-term local IGF-1 and insulin delivery to induce adipogenic differentiation to mature lipid-containing adipocytes from non-adipocyte cell pools (e.g., ASCs) that were administered directly to the deep muscular fascia of the rat abdominal wall.

In addition to PLGA microspheres, the use of extracellular matrix (ECM) particles for injectable systems for adipose tissue engineering has been studied [139,148]. We have previously reported the assessment of ASC attachment, proliferation, and differentiation on gelatinous microparticles, termed CultiSphers [139]. These results demonstrated the potential of using biodegradable particles as cell carriers for soft tissue repair.

## 5. Conclusions

Injectable scaffolds are promising substrates for tissue engineering with the advantage that drugs and cells can be readily integrated into the gelling matrix. Many efforts have been developed to improve injectable hydrogels and thus, support the development of more natural and functional tissues. The success of injectable tissue constructs is highly dependent on the design of the hydrogel scaffolds including physical, chemical and biological properties. An ideal injectable hydrogel would potentially mimic many roles of ECM found in tissues, resulting in the coexistence of both physical and chemical gels. Current biomaterials are unable to meet all the design parameters simultaneously (e.g., degradation, biocompatibility or mechanical properties). Furthermore, injectable hydrogel development will likely have a significant impact on the advancement of tissue engineering. An objective in future work is to design bioactive materials that would be readily injectable at or below room temperature, would form gels with relatively appropriate biodegradable properties under physiological conditions, and would support cell induction. Novel crosslinking methods should be developed, both to enhance the material biocompatibility as well as control the mechanical properties. In addition, cell induction ligands such as growth factors and genes can be incorporated into the injectable scaffolds such that specific signals could be delivered in an appropriate spatial and temporal manner.
